# Wealth and cardiovascular health: a cross-sectional study of wealth-related inequalities in the awareness, treatment and control of hypertension in high-, middle- and low-income countries

**DOI:** 10.1186/s12939-016-0478-6

**Published:** 2016-12-08

**Authors:** Benjamin Palafox, Martin McKee, Dina Balabanova, Khalid F. AlHabib, Alvaro Jr Avezum, Ahmad Bahonar, Noorhassim Ismail, Jephat Chifamba, Clara K. Chow, Daniel J. Corsi, Gilles R. Dagenais, Rafael Diaz, Rajeev Gupta, Romaina Iqbal, Manmeet Kaur, Rasha Khatib, Annamarie Kruger, Iolanthe Marike Kruger, Fernando Lanas, Patricio Lopez-Jaramillo, Fu Minfan, Viswanathan Mohan, Prem K. Mony, Aytekin Oguz, Lia M. Palileo-Villanueva, Pablo Perel, Paul Poirier, Sumathy Rangarajan, Lei Rensheng, Annika Rosengren, Biju Soman, David Stuckler, S. V. Subramanian, Koon Teo, Lungiswa P. Tsolekile, Andreas Wielgosz, Peng Yaguang, Karen Yeates, Mo Yongzhen, Khalid Yusoff, Rita Yusuf, Afzalhussein Yusufali, Katarzyna Zatońska, Salim Yusuf

**Affiliations:** 1 ECOHOST – The Centre for Health and Social Change, London School of Hygiene & Tropical Medicine, 15-17 Tavistock Place, London, WC1H 9SH UK; 2 Department of Cardiac Sciences, King Fahad Cardiac Center, College of Medicine, King Saud University, Riyadh, Saudi Arabia; 3 Dante Pazzanese Institute of Cardiology, São Paulo, Brazil; 4 Hypertension Research Center, Cardiovascular Research Institute, Isfahan University of Medical Sciences, Isfahan, Iran; 5 Faculty of Medicine, University Kebangsaan Malaysia, Kuala Lumpur, Malaysia; 6 Physiology Department, College of Health Sciences, University of Zimbabwe, Harare, Zimbabwe; 7 The George Institute for Global Health, Sydney Medical School, The University of Sydney, Sydney, NSW Australia; 8 Ottawa Hospital Research Institute, Ottawa, Canada; 9 Quebec Heart and Lung University Institute, Quebec City, QC Canada; 10 Estudios Clinicos Latinoamerica, Rosario, Santa Fe Argentina; 11 Eternal Heart Care Centre and Research Institute, Jaipur, Rajasthan India; 12 Departments of Community Health Sciences and Medicine, Aga Khan University, Karachi, Pakistan; 13 School of Public Health, Post Graduate Institute of Medical Education and Research, Chandigarh, India; 14 Population Health Research Institute, McMaster University, Hamilton, Ontario Canada; 15 Africa Unit for Transdisciplinary Health Research and Medical Research Council Research Unit for Hypertension and Cardiovascular Disease, Faculty of Health Sciences, North-West University, Potchefstroom, South Africa; 16 Africa Unit for Transdisciplinary Health Research, Faculty of Health Sciences, North-West University, Potchefstroom, South Africa; 17 Universidad de la Frontera, Temuco, Chile; 18 Research Institute, Fundacion Oftalmologica de Santander; and Medical School, University of Santander, Floridablanca, Bucaramanga Colombia; 19 Daxing Health Center, Shenyang City, Liaoning Province China; 20 Madras Diabetes Research Foundation, Chennai, India; 21 St John’s Medical College & Research Institute, Bangalore, India; 22 Department of Internal Medicine, Istanbul Medeniyet University, Istanbul, Turkey; 23 College of Medicine, University of the Philippines Manila, Manila, Philippines; 24 World Heart Federation, Geneva, Switzerland; 25 The London School of Hygiene & Tropical Medicine, London, UK; 26 Faculté de Pharmacie, Université Laval Institut Universitaire de Cardiologie et de Pneumologie de Québec, Québec, Canada; 27 Population Health Research Institute, Hamilton Health Sciences and McMaster University, Hamilton, Ontario Canada; 28 Center for Disease Control & Prevention, Nanchang City, Jiangxi Province China; 29 Department of Molecular and Clinical Medicine, Sahlgrenska Academy, Sahlgrenska University Hospital/Östra Hospital, Göteborg, Sweden; 30 Health Action by People, Thiruvananthapuram, and Achutha Menon Centre for Health Science Studies, Sree Chitra Institute for Medical Sciences & Technology, Trivandrum, Kerala India; 31 Department of Sociology, University of Oxford, Oxford, UK; 32 Department of Social and Behavioral Sciences, Harvard University, Boston, MA USA; 33 University of the Western Cape, Bellville, Western Province South Africa; 34 The Ottawa Hospital, Ottawa, Ontario Canada; 35 Medical Research & Biometrics Center, National Center for Cardiovascular Diseases, FuWai Hospital, Beijing, China; 36 Department of Medicine, Queen’s University, Kingston, Canada; 37 Institute of Geriatrics, Nanjing City, Jiangsu Province China; 38 UCSI University, Kuala Lumpur, Malaysia; 39 Universiti Teknologi MARA, Selayang, Selangor Malaysia; 40 Independent University, Dhaka, Bangladesh; 41 Hatta Hospital, Dubai Health Authority, Dubai, United Arab Emirates; 42 Department of Social Medicine, Wroclaw Medical University, Wroclaw, Poland

**Keywords:** Global health, Hypertension, Socioeconomic factors, Healthcare disparities

## Abstract

**Background:**

Effective policies to control hypertension require an understanding of its distribution in the population and the barriers people face along the pathway from detection through to treatment and control. One key factor is household wealth, which may enable or limit a household’s ability to access health care services and adequately control such a chronic condition. This study aims to describe the scale and patterns of wealth-related inequalities in the awareness, treatment and control of hypertension in 21 countries using baseline data from the Prospective Urban and Rural Epidemiology study.

**Methods:**

A cross-section of 163,397 adults aged 35 to 70 years were recruited from 661 urban and rural communities in selected low-, middle- and high-income countries (complete data for this analysis from 151,619 participants). Using blood pressure measurements, self-reported health and household data, concentration indices adjusted for age, sex and urban-rural location, we estimate the magnitude of wealth-related inequalities in the levels of hypertension awareness, treatment, and control in each of the 21 country samples.

**Results:**

Overall, the magnitude of wealth-related inequalities in hypertension awareness, treatment, and control was observed to be higher in poorer than in richer countries. In poorer countries, levels of hypertension awareness and treatment tended to be higher among wealthier households; while a similar pro-rich distribution was observed for hypertension control in countries at all levels of economic development. In some countries, hypertension awareness was greater among the poor (Sweden, Argentina, Poland), as was treatment (Sweden, Poland) and control (Sweden).

**Conclusion:**

Inequality in hypertension management outcomes decreased as countries became richer, but the considerable variation in patterns of wealth-related inequality - even among countries at similar levels of economic development - underscores the importance of health systems in improving hypertension management for all. These findings show that some, but not all, countries, including those with limited resources, have been able to achieve more equitable management of hypertension; and strategies must be tailored to national contexts to achieve optimal impact at population level.

**Electronic supplementary material:**

The online version of this article (doi:10.1186/s12939-016-0478-6) contains supplementary material, which is available to authorized users.

## Background

In 2013 the World Health Organization (WHO) published its Global Action Plan for the Prevention and Control of Non-Communicable Diseases (NCDs), with one of nine voluntary targets to reduce preventable deaths from cardiovascular diseases (CVD) through increased use of secondary prevention measures and improvements in hypertension control [1]. The Sustainable Development Goals have subsequently reinforced the need to tackle NCDs, but there are many barriers to be overcome [2].

One such barrier is a lack of understanding of the scale and nature of the gaps in care, all along the pathway from early detection of hypertension to treatment and control, including differences among population groups within individual countries. Among the many studies on hypertension management, only a minority examine the entire pathway, and even fewer examine inequalities at each stage. Among those that do, the majority are from high-income country settings. The Prospective Urban Rural Epidemiology (PURE) study, a large multi-country longitudinal study of NCD risk factors and outcomes, has revealed marked differences in hypertension prevalence, awareness, treatment and control by age, gender, and education level in countries at all income levels, and between urban and rural locations [3]. Yet, other than this and a few other exceptions [4–6], comparative studies of inequalities in the treatment and control of hypertension are sparse.

Those studies that exist have largely focused on education and ethnicity as measures of socio-economic status (SES) yet a comprehensive understanding also requires information on measures more directly related to contemporary economic status, such as household income and wealth. Such measures more directly reflect a household’s command over its resources and thus, potentially, the ability to obtain health care. Studies that have examined inequalities in hypertension awareness, treatment or control associated with household income or wealth have typically done so by comparing outcomes across wealth quintiles. In one multi-country study, for example, those from richer households in China, Ghana, India, Mexico, Russia and South Africa tended to be more likely to have their hypertension controlled [5]; however, the magnitude of this inequality cannot be easily quantified or summarized using this approach, or compared across countries.

In this paper we undertake further analysis of data from the PURE study and go beyond our earlier work in two ways: first by characterizing the scale of wealth-related inequalities in hypertension awareness, treatment and control using a summary measure that can reliably be compared across countries and outcomes; and second, by examining 21 individual countries rather than groups of countries at different levels of development.

## Methods

### Study design and participants

This cross-sectional analysis uses baseline data from individuals enrolled in the PURE cohort study, which seeks to determine the relationship between the burden of NCDs and a range of individual, household, and community characteristics. Its methods have been described elsewhere [7, 8]. Briefly, participants were recruited from communities in 21 countries, purposefully selected to capture wide variation in sociocultural diversity and economic development, as defined by the World Bank in 2006 [9]. Low-income countries include Bangladesh, India, Pakistan, Tanzania, and Zimbabwe; lower middle-income countries include China, Colombia, Iran, Occupied Palestinian Territory (OPT), and the Philippines; upper middle-income countries include Argentina, Brazil, Chile, Malaysia, Poland, South Africa, and Turkey; and high-income countries were Canada, Saudi Arabia, Sweden and the United Arab Emirates (UAE). The timing of data collection in each country is shown in Additional file 1.

Within each country, participants were selected from communities in both rural and urban areas. Communities were defined as groups of people who reside within a specific geographic area and who were generally expected to have similar characteristics (e.g. culture, socioeconomic status, use of amenities, goods, and services). Existing administrative boundaries, such as village limits or postal code areas, or physical features (e.g. area bounded by selected streets) were used to define urban communities, while rural communities were defined as villages or postal code areas located at least 50 km away from an urban center. Within strata, communities were drawn from areas of varying income levels. Sampling within participating communities sought to recruit an unbiased sample of households (Additional file 2 provides supplemental information on sampling).

Eligible households had at least one member aged 35 to 70 years intending to stay at that address for another 4 years. For practical reasons the goal was not to undertake proportionate sampling, but rather, to show economic and sociocultural diversity and include sites where investigators were committed to collecting high-quality data at a modest budget and following up participants over a number of years. However, those included are broadly comparable to the populations of the countries in which they reside in terms of risk factors and mortality (Additional file 3 provides supplemental information on the characteristics of the PURE samples) [10, 11]. Eligible participants between the ages of 35 and 70 years who provided written informed consent completed a standardized questionnaire on medical history, individual- and household-level risk factors, and underwent a basic physical examination as described in the INTERHEART study [12] (Additional file 4 provides supplemental information on these procedures). The PURE study was approved by research ethics committees in the participating countries.

### Measurement of health-related outcomes and participant characteristics

Sitting blood pressure was measured twice by trained research assistants following a standardized procedure using an Omron digital blood pressure measuring device (Omron HEM-757), as recommended by the WHO STEPwise approach to Surveillance (STEPS) [13]. Subjects listed all current prescription medications. This information provided the proportion of participants with hypertension who were aware of their condition and who were receiving treatment. In the main analyses, control was the proportion of participants with hypertension who had an average systolic and diastolic blood pressure of less than 140/90 mm Hg, also as recommended by the WHO STEPS protocol [13] and in many current guidelines [14]. We report three standard definitions of hypertension (Table 1) to enable comparisons with other research [15], but use the standard ‘lower threshold’ definition to define the denominator in the main analyses.

**Table 1:** Definitions of hypertension

Standard lower threshold definition^a^	Subjects reported having been diagnosed with hypertension by a health care professional *and* receiving blood pressure-lowering medication, or if they had an average sitting systolic blood pressure of at least 140 mm Hg, diastolic blood pressure of at least 90 mm Hg, or both.
Alternative lower threshold definition	Subjects reported having been diagnosed with hypertension by a health care professional *or* receiving blood pressure-lowering medication, or if they had an average systolic blood pressure of at least 140 mm Hg, diastolic blood pressure of at least 90 mm Hg, or both.
Alternative ‘higher threshold’ definition	Subjects reported having been diagnosed with hypertension by a health care professional *and* receiving blood pressure-lowering medication, or if they had an average blood pressure above the threshold of 160/100 mm Hg.

^a^The paper reports three definitions to enable comparisons with other research. However, the main analyses by hypertension management outcome use the first of these to define the denominator

Following the Demographic and Health Surveys (DHS), the questionnaire collected an extensive range of socio-demographic data, including household possessions: electricity supply, automobile, computer, television, motorbike, livestock, fridge, other four-wheel vehicle, washing machine, stereo, bicycle, kitchen mixer, telephone, land/real estate and kitchen window [16]. Within each country, these were used to generate an asset-based wealth index, based on that used in the DHS and using principal components analysis, which places surveyed households on a continuous scale of relative wealth from poorest to richest [17]. This approach is designed to permit meaningful comparisons of observed inequalities across countries at different levels of development [18]. The continuous score was divided into country-specific wealth quintiles that reflect the distribution of household wealth within each of the country cohorts. Additional file 5 provides further details on the asset-based wealth index. Age was calculated in years from date of birth, or self-reported age in years when date of birth was unknown.

### Analysis of wealth-related differences

For each country, we used the continuous household wealth score to generate a summary measure of the degree of wealth-related differences for each hypertension outcome. We estimated the Wagstaff concentration index indirectly standardized for age, gender and urban/rural location derived using the convenient regression approach to produce point estimates and 95% confidence intervals corrected for within-cluster correlation at community level obtained using the delta method [19–21]. As the binary outcomes were coded as 0 for no and 1 for yes, index values were normalized by dividing by 1 minus the mean [22].

The value of the index ranges from –1 to 1, where a negative value indicates a disproportionate concentration of the outcome among the poor, and a positive value indicates a disproportionate concentration of the outcome among the rich. The index is zero when there is no wealth-related inequality. Thus, for the different outcomes, a positive index means that the probability of having hypertension and being aware of it, or having it treated or controlled, is higher among the rich (i.e. the outcomes are ‘pro-rich’). The magnitude of the concentration index can be used to give a relative sense of the degree of inequality in either direction (i.e. closer to 0 = more equal).

We present the concentration index as our core measure of relative wealth-related inequality; however, we also illustrate absolute inequalities for each outcome in two ways. Within each country, we used mixed-effects logistic regression to analyze data on those with hypertension, as defined above, and generated estimates of three prevalence outcomes: being aware of one’s hypertension diagnosis, being treated for hypertension, and having controlled hypertension. Although basic logit models without random effects produced a better fit in some countries with few communities, the mixed-effects model enabled us to apply a consistent approach, thus enabling comparability. The estimates were adjusted for age, gender, urban/rural location, and a random effect for community. These were plotted by wealth quintile to show the patterns at each stage of the treatment pathway, and the difference in prevalence between the poorest and richest quintiles, with 95% confidence intervals and p-values, to quantify absolute inequalities. Although this approach is intuitive, we also present the slope index of inequality for each outcome generated using the same mixed-effects logistic regression framework above. Similar to the concentration index, this alternative summary measure uses data across the entire sample, but examines absolute, rather than relative, inequality [23]. We used Stata v.13 for all analyses [24].

Finally, to identify potential correlates of the magnitude of wealth-related inequalities we used log-linear regression trend lines to compare hypertension management outcomes with economic development of each country, measured by its 2012 per capita Gross National Product (in US dollars) [9].

## Results

### Participant characteristics

Of the 163,397 participants who were enrolled in the PURE cohort across the 21 study countries and 661 communities, the personal and household information required for the current analyses was available from 151,619 participants. Table 2 shows the characteristics of each country sample. Under half (46%) lived in rural communities, 58% were women, and the mean age was 50.6 years.

**Table 2:** Characteristics of PURE cohort, by country (ordered by 2006 GDP)

2006 World Bank country income group			Participants						
	Country	No. of Communities	Number	No. Rural (%)		No. Women (%)		Mean Age in years (SD)	
High income	Canada	77	10412	2527	(24.3)	5588	(53.7)	53.4	(9.2)
	Sweden	24	4150	902	(21.7)	2192	(52.8)	52.7	(9.0)
	UAE	3	918	429	(46.7)	653	(71.1)	49.2	(10.2)
	Saudi Arabia	18	1729	186	(10.8)	734	(42.5)	46.5	(9.0)
Upper-middle income	Argentina	21	7497	3897	(52.0)	4607	(61.5)	51.1	(9.9)
	Brazil	14	5581	1302	(23.3)	3086	(55.3)	52.2	(9.4)
	Chile	5	3270	694	(21.2)	2158	(66.0)	51.9	(9.9)
	Malaysia	36	11825	7024	(59.4)	6736	(57.0)	51.3	(9.8)
	Poland	4	2029	825	(40.7)	1274	(62.8)	54.6	(9.8)
	South Africa	11	3252	1766	(54.3)	2213	(68.1)	49.9	(10.3)
	Turkey	38	4060	1426	(35.1)	2459	(60.6)	50.0	(9.1)
Lower-middle income	China	110	46751	23558	(50.4)	27318	(58.4)	51.0	(10.0)
	Philippines	2	1671	630	(37.7)	1125	(67.3)	51.8	(9.7)
	Colombia	60	7506	4036	(53.8)	4819	(64.2)	50.7	(9.8)
	Iran	20	6013	2982	(49.6)	3137	(52.2)	48.5	(9.2)
	OPT	39	1563	663	(42.4)	780	(49.9)	49.3	(10.0)
Low income	Bangladesh	56	2747	1437	(52.3)	1504	(54.8)	46.0	(9.4)
	India	97	27458	14300	(52.1)	15507	(56.5)	48.7	(10.5)
	Pakistan	4	1294	373	(28.8)	674	(52.1)	47.4	(8.7)
	Zimbabwe	3	822	568	(69.1)	590	(71.8)	52.4	(10.1)
	Tanzania	19	1071	420	(39.2)	833	(77.8)	49.9	(11.3)
	Total	661	151619	69945	(46.1)	87987	(58.0)	50.6	(10.0)

*OPT* occupied Palestinian territory, *UAE* United Arab Emirates, *SD* standard deviation, *GDP* gross domestic product

Table 3 shows the percentage of those with hypertension who were aware, treated and controlled (both lower threshold definitions). Corresponding figures for the higher threshold definition are shown in Additional file 6. Nearly half (41%) of participants either self-reported a history of hypertension and current use of antihypertensive medications, or had an average blood pressure greater or equal to 140/90 mm Hg. Among those with hypertension, 47% were aware of their diagnosis; 41% were being treated; and 13% had their hypertension controlled. Additional file 7: Figure S1 illustrates patterns of inequalities in adjusted hypertension prevalence (standard lower threshold definition) by wealth quintile and measured using concentration indices. Hypertension was significantly more common among the richer households in South Africa, the Philippines and in all low-income countries. It was more common among poorer households in Canada, Argentina, Malaysia, Turkey and Iran. Elsewhere the differences were not significant.

**Table 3:** Hypertension burden and management in the PURE cohort, by country (ordered by 2006 GDP)

	Participants							Hypertensive participants – Standard lower threshold ^a^								
Country	Number	No. with Hypertension – Standard lower threshold ^a^ (%) [95%CI]			No. with Hypertension – Alternative lower threshold^a^ (%) [95%CI]			No. Aware (%) [95%CI]			No. Treated (%) [95%CI]			No. Controlled (%) [95%CI]		
Canada	10412	3908	(37.5)	[35.3–39.8]	4347	(41.7)	[39.4–44.1]	2157	(55.2)	[53.3–57.0]	2112	(54.0)	[52.2–55.9]	968	(24.8)	[23.2–26.4]
Sweden	4150	1923	(46.3)	[44.3–48.4]	2008	(48.4)	[46.1–50.6]	696	(36.2)	[32.0–40.6]	608	(31.6)	[26.8–36.8]	169	(8.8)	[7.5–10.3]
UAE	918	477	(52.0)	[48.7–55.2]	487	(53.1)	[49.8–56.3]	247	(51.8)	[47.3–56.3]	240	(50.3)	[45.8–54.8]	63	(13.2)	[10.4–16.6]
Saudi Arabia	1729	515	(29.8)	[27.1–32.6]	546	(31.6)	[29.0–34.3]	319	(61.9)	[56.8–66.9]	309	(60.0)	[55.2–64.6]	169	(32.8)	[28.5–37.5]
Argentina	7497	3809	(50.8)	[49.1–52.5]	4045	(54.0)	[52.5–55.4]	2073	(54.4)	[51.7–57.2]	1924	(50.5)	[47.8–53.3]	569	(14.9)	[12.8–17.4]
Brazil	5581	2938	(52.6)	[51.0–54.3]	3096	(55.5)	[54.2–56.7]	1896	(64.5)	[61.9–67.1]	1838	(62.6)	[60.0–65.1]	685	(23.3)	[21.7–25.0]
Chile	3270	1530	(46.8)	[42.6–51.0]	1664	(50.9)	[46.5–55.2]	890	(58.2)	[53.8–62.4]	798	(52.2)	[46.6–57.6]	332	(21.7)	[18.0–25.9]
Malaysia	11825	5509	(46.6)	[43.9–49.3]	5894	(49.8)	[47.3–52.3]	2648	(48.1)	[45.3–50.9]	2272	(41.2)	[38.1–44.5]	690	(12.5)	[10.9–14.3]
Poland	2029	1368	(67.4)	[63.6–71.0]	1412	(69.6)	[65.7–73.2]	739	(54.0)	[35.0–71.9]	706	(51.6)	[33.4–69.4]	151	(11.0)	[5.4–21.1]
South Africa	3252	1857	(57.1)	[45.4–68.1]	1975	(60.7)	[50.1–70.5]	580	(31.2)	[20.3–44.7]	616	(33.2)	[26.3–40.8]	117	(6.3)	[3.9–9.9]
Turkey	4060	1595	(39.3)	[36.3–42.3]	1770	(43.6)	[40.6–46.6]	910	(57.1)	[53.1–60.9]	808	(50.7)	[46.6–54.8]	330	(20.7)	[16.9–25.0]
China	46751	19471	(41.6)	[39.2–44.1]	20367	(43.6)	[41.2–46.0]	8114	(41.7)	[37.5–46.0]	6557	(33.7)	[29.3–38.4]	1556	(8.0)	[5.9–10.7]
Philippines	1671	855	(51.2)	[43.3–59.0]	891	(53.3)	[45.2–61.3]	466	(54.5)	[29.1–77.8]	394	(46.1)	[21.0–73.3]	115	(13.5)	[3.8–37.7]
Colombia	7506	2817	(37.5)	[35.7–39.4]	3110	(41.4)	[39.8–43.0]	1461	(51.9)	[48.7–55.0]	1309	(46.5)	[43.2–49.7]	484	(17.2)	[15.1–19.5]
Iran	6013	1598	(26.6)	[23.6–29.8]	1939	(32.2)	[29.3–35.4]	841	(52.6)	[45.7–59.5]	816	(51.1)	[44.5–57.6]	293	(18.3)	[13.4–24.6]
OPT	1563	591	(37.8)	[34.1–41.6]	657	(42.0)	[38.5–45.6]	347	(58.7)	[51.8–65.3]	345	(58.4)	[52.4–64.1]	130	(22.0)	[16.5–28.6]
Bangladesh	2747	1080	(39.3)	[36.1–42.7]	1154	(42.0)	[38.7–45.4]	261	(24.2)	[20.3–28.5]	174	(16.1)	[13.1–19.7]	43	(4.0)	[2.6–6.0]
India	27458	8473	(30.9)	[28.5–33.4]	9443	(34.4)	[31.7–37.2]	3565	(42.1)	[38.1–46.2]	2843	(33.6)	[29.9–37.4]	1159	(13.7)	[11.1–16.7]
Pakistan	1294	435	(33.6)	[25.8–42.4]	565	(43.7)	[40.2–47.1]	206	(47.4)	[39.2–55.6]	162	(37.2)	[28.7–46.6]	76	(17.5)	[12.9–23.2]
Zimbabwe	822	453	(55.1)	[37.0–72.0]	515	(62.7)	[44.8–77.6]	223	(49.2)	[31.3–67.4]	119	(26.3)	[17.1–38.0]	36	(7.9)	[4.9–12.6]
Tanzania	1071	410	(38.3)	[34.7–42.0]	439	(41.0)	[37.1–45.0]	45	(11.0)	[7.4–15.9]	12	(2.9)	[2.0–4.3]	2	(0.5)	[0.1–1.7]
Total	151619	61612	(40.6)		66324	(43.7)		28684	(46.6)		24962	(40.5)		8137	(13.2)	

*OPT* occupied Palestinian territory, *UAE* United Arab Emirates, *SD* standard deviation, *CI* confidence interval, *GDP* gross domestic product. Notes: ^a^Hypertension standard lower threshold definition: systolic blood pressure ≥140 mm Hg or diastolic blood pressure ≥90 mm Hg or who report currently taking medication; Hypertension alternative lower threshold: in addition to those under the standard lower threshold definition, also includes those who report having been diagnosed with hypertension by a health professional. Additional file 6 reports additional indicators using the higher threshold definition of hypertension

### Gaps in the hypertension management pathway within countries

In all countries, a substantial number of hypertensive subjects were unaware of their condition (Table 3) and, of those who were aware, only a proportion was receiving treatment. For example, in Canada, an estimated 55.2% of patients were aware they had hypertension, and, among those, 97.9% were treated. Of these, only 45.8% were controlled, revealing that, in total, an estimated 24.8% of hypertensive patients were adequately managed. In India, the corresponding numbers were 42.1, 79.7, and 40.8%, respectively, so that only 13.7% of those with hypertension were adequately managed. The highest level of control was seen in Saudi Arabia, with the lowest in Tanzania.

### Wealth-related inequalities in the hypertension management pathway within countries

**Fig. 1:**
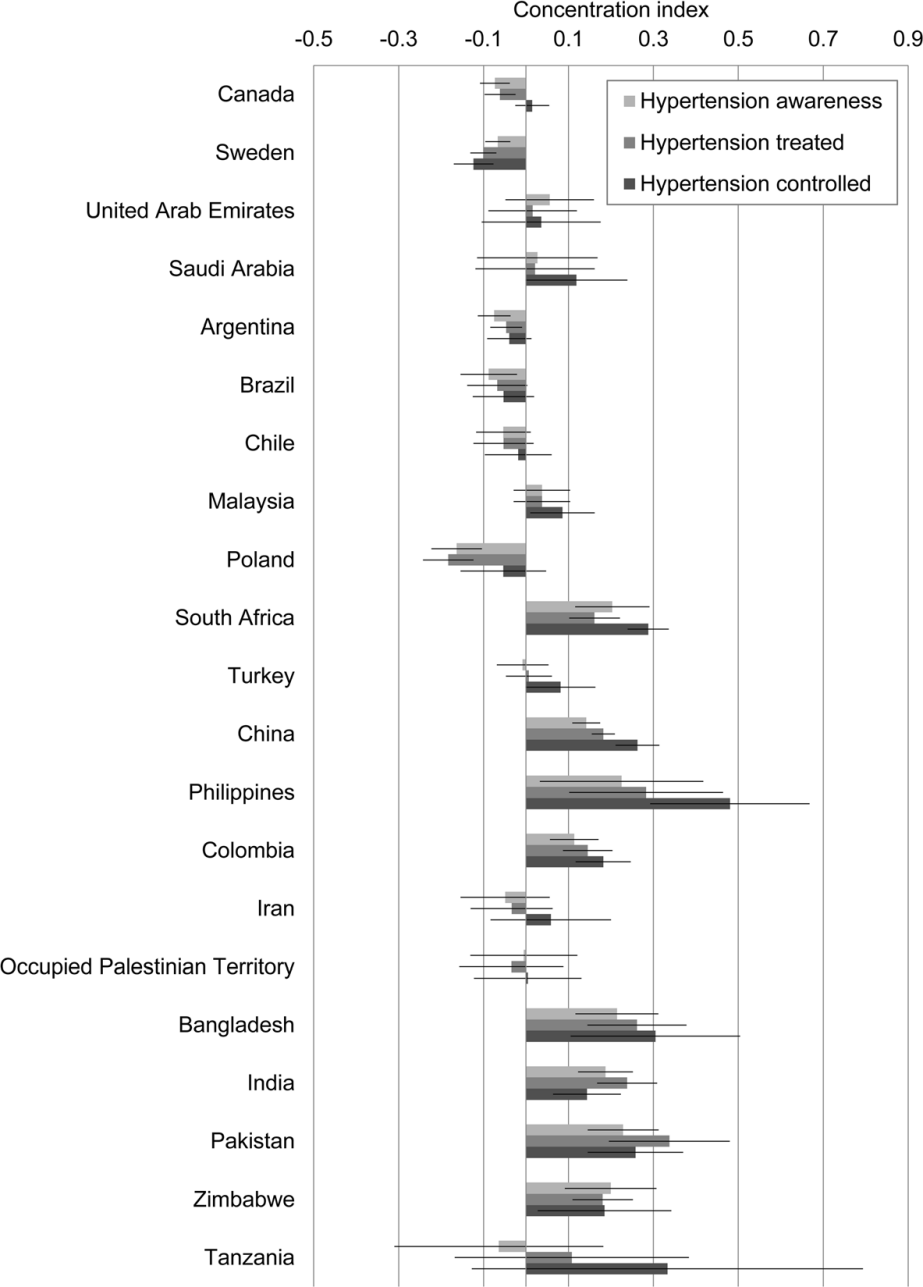
Magnitude of wealth-related inequalities in hypertension awareness, treatment and control measured by concentration index with 95% confidence interval

The concentration indices, standardized for age, sex and urban-rural location (Fig. 1), show that those with hypertension from richer households were significantly more likely to be aware than those from poorer households in most lower-income countries. In contrast, awareness was greater among poorer households in Canada, Sweden, Argentina, Brazil and Poland, although the magnitude of these inequalities was relatively small. The inequalities in hypertension awareness were not statistically significant in the remaining countries. Treatment rates were higher among richer households in South Africa, China, the Philippines, Colombia, Bangladesh, India, Pakistan and Zimbabwe. They were higher among poorer households in Canada, Sweden and Poland.

Only in Sweden was control better among poorer participants. In Saudi Arabia, Malaysia and Turkey there was no significant inequality in awareness and treatment, but control was significantly better among richer households. In China, the Philippines and Colombia, inequalities appear to intensify along each stage of the management pathway, but many of these trends were not statistically significant. Further analyses of absolute inequalities are consistent with these findings, and are shown by wealth quintile in Table 4 and Additional file 8: Figure S2, and by slope index of inequality in Additional file 9: Figure S3.

**Table 4:** Difference between the poorest (Q1) and richest (Q5) wealth quintiles in adjusted estimates^a^ for hypertension burden and management in the PURE cohort, by country (ordered by 2006 GDP)

Country	Hypertension % of all participants					Hypertension awareness % of hypertensive participants					Hypertension treatment % of hypertensive participants					Hypertension control % of hypertensive participants				
	Q1	Q5	Difference [95% CI]		*p*-value	Q1	Q5	Difference [95% CI]		*p*-value	Q1	Q5	Difference [95% CI]		*p*-value	Q1	Q5	Difference [95% CI]		*p*-value
Canada	38.5	34.2	−4.3	[-8.0--0.7]	0.019	58.6	52.1	−6.5	[-12.5--0.6]	0.031	55.6	51.8	−3.7	[-9.8–2.4]	0.229	21.8	24.9	3.0	[-2.0–8.0]	0.240
Sweden	46.5	46.3	−0.2	[-5.4–5.0]	0.941	37.8	34.1	−3.7	[-10.8–3.4]	0.305	34.1	26.8	−7.3	[-14.1--0.5]	0.034	9.7	6.9	−2.8	[-6.7–1.1]	0.161
UAE	46.5	50.7	4.2	[-7.3–15.6]	0.476	45.1	55.9	10.9	[-4.6–26.3]	0.168	46.8	52.9	6.1	[-9.5–21.6]	0.444	7.2	12.2	5.0	[-3.9–13.9]	0.271
Saudi Arabia	28.2	26.9	−1.3	[-8.6–6.0]	0.730	70.2	70.4	0.2	[-14.0–14.3]	0.983	68.4	66.4	−2.0	[-16.7–12.7]	0.788	23.8	37.5	13.8	[0.2–27.4]	0.047
Argentina	51.2	46.7	−4.6	[-8.6--0.5]	0.028	57.9	52.7	−5.2	[-10.5–0.2]	0.060	49.9	49.3	−0.6	[-6.1–4.8]	0.816	13.1	12.5	−0.6	[-4.0–2.8]	0.734
Brazil	53.9	52.7	−1.2	[-6.2–3.7]	0.634	67.7	60.4	−7.3	[-13.7--0.9]	0.025	67.1	62.3	−4.8	[-11.7–2.0]	0.168	26.1	22.2	−3.9	[-10.1–2.3]	0.213
Chile	45.2	45.3	0.1	[-6.9–7.1]	0.981	60.6	60.0	−0.6	[-10.2–9.0]	0.902	50.3	51.9	1.6	[-8.7–11.9]	0.757	16.4	19.0	2.6	[-4.6–9.8]	0.473
Malaysia	42.8	42.7	−0.2	[-3.5–3.1]	0.926	44.4	51.0	6.6	[1.6–11.6]	0.010	36.6	41.5	4.9	[0.0–9.8]	0.052	9.8	12.2	2.5	[-0.6–5.5]	0.110
Poland	69.2	73.8	4.7	[-3.3–12.6]	0.248	54.2	46.0	−8.3	[-18.9–2.4]	0.128	53.4	42.1	−11.3	[-22.0--0.6]	0.038	7.3	7.7	0.4	[-4.6–5.5]	0.869
South Africa	59.9	59.3	−0.6	[-7.2–6.0]	0.855	21.4	34.1	12.7	[5.0–20.4]	0.001	27.7	38.1	10.4	[2.6–18.1]	0.009	2.0	9.3	7.2	[3.8–10.7]	0.000
Turkey	37.5	31.4	−6.1	[-11.3--0.8]	0.023	56.3	57.1	0.7	[-7.8–9.3]	0.864	49.4	50.4	1.0	[-7.6–9.6]	0.820	17.6	22.2	4.5	[-2.2–11.2]	0.185
China	40.8	38.4	−2.4	[-4.3--0.6]	0.009	39.2	44.8	5.7	[2.8–8.5]	0.000	26.7	34.0	7.3	[4.7–9.9]	0.000	4.1	7.7	3.6	[2.2–4.9]	0.000
Philippines	43.0	58.6	15.6	[4.1–27.1]	0.008	44.7	69.7	25.0	[9.1–40.9]	0.002	34.1	64.5	30.4	[14.4–46.3]	0.000	4.8	26.1	21.3	[9.1–33.5]	0.001
Colombia	34.8	32.9	−1.9	[-6.6–2.7]	0.416	46.4	59.0	12.6	[4.5–20.7]	0.002	38.4	53.6	15.3	[7.1–23.4]	0.000	10.9	19.5	8.6	[2.7–14.6]	0.004
Iran	25.3	20.2	−5.1	[-9.2--1.0]	0.015	53.3	58.1	4.8	[-5.3–14.8]	0.350	49.8	55.9	6.0	[-4.2–16.2]	0.246	16.0	18.2	2.2	[-5.0–9.3]	0.554
OPT	37.8	33.7	−4.1	[-13.1–4.8]	0.368	62.2	52.1	−10.1	[-25.1–4.9]	0.188	60.0	51.3	−8.7	[-23.5–6.1]	0.250	17.3	13.3	−3.9	[-13.8–5.9]	0.431
Bangladesh	31.8	52.0	20.2	[13.8–26.6]	0.000	14.0	24.9	10.9	[2.0–19.8]	0.016	8.8	21.2	12.5	[4.5–20.4]	0.002	1.7	4.9	3.2	[-0.6–7.0]	0.101
India	13.6	35.4	21.9	[19.2–24.6]	0.000	27.2	48.0	20.8	[16.3–25.3]	0.000	20.9	43.0	22.1	[17.7–26.6]	0.000	8.9	15.0	6.1	[3.2–9.1]	0.000
Pakistan	29.8	31.7	1.8	[-11.2–14.8]	0.784	27.9	56.2	28.3	[8.8–47.9]	0.005	15.7	50.1	34.4	[16.7–52.2]	0.000	9.9	26.2	16.3	[1.3–31.3]	0.033
Zimbabwe	57.9	64.3	6.4	[-10.2–23.1]	0.449	43.1	51.1	8.0	[-15.8–31.8]	0.511	19.5	25.8	6.3	[-11.9–24.5]	0.497	4.4	8.0	3.7	[-6.6–13.9]	0.485
Tanzania^+^	34.7	40.0	5.3	[-6.7-17.2]	0.389	9.7	5.1	-4.5	[-13.7-4.7]	0.338	3.0	3.9	0.9	[-5.7-7.5]	0.790					

*Q1* poorest wealth quintile, *Q5* richest wealth quintile, *UAE* United Arab Emirates, *OPT* occupied Palestinian territory, *CI* confidence interval. Note: ^a^Indirectly standardized for age and sex, and controlled for urban-rural location. ^+^ Adjusted estimates for hypertension control by wealth quintile in Tanzania could not be obtained due to the low number of positive outcomes

### Hypertension management and economic development

**Fig. 2:**
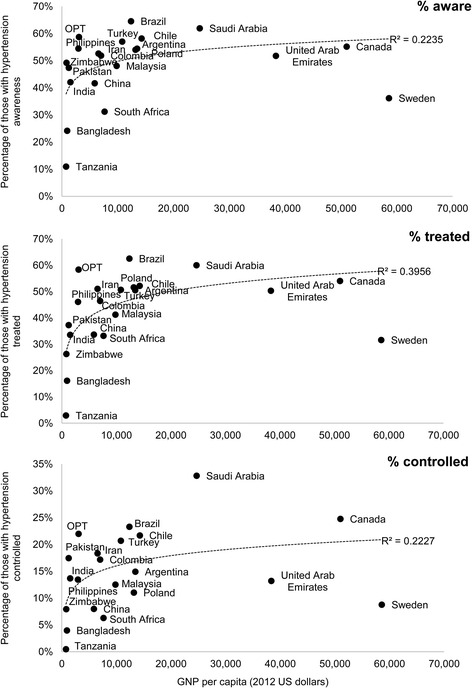
Association between outcomes of hypertension awareness, treatment and control, and economic development measured as GNP per capita (2012 US dollars)

It might be expected that hypertension detection, treatment and control would be better in richer countries, which can afford strong health systems. Figure 2 examines the association of hypertension management outcomes with Gross National Product, with a trend line based on the log values of the variables. The lowest levels of awareness were seen in the poorest countries; yet this is not inevitable as in some poor countries, levels of awareness were high. Awareness was lower than expected given national income in Sweden and South Africa, but was higher than expected in Brazil, Saudi Arabia, OPT and the Philippines. A similar pattern is seen with levels of treatment, although there was a particularly large gap between awareness and treatment in low-income countries and in China and the Philippines. Control rates were low everywhere, but again some of the lowest levels are seen in the poorest countries, with Sweden performing unexpectedly poorly.

### Wealth-related inequalities and economic development

**Fig. 3:**
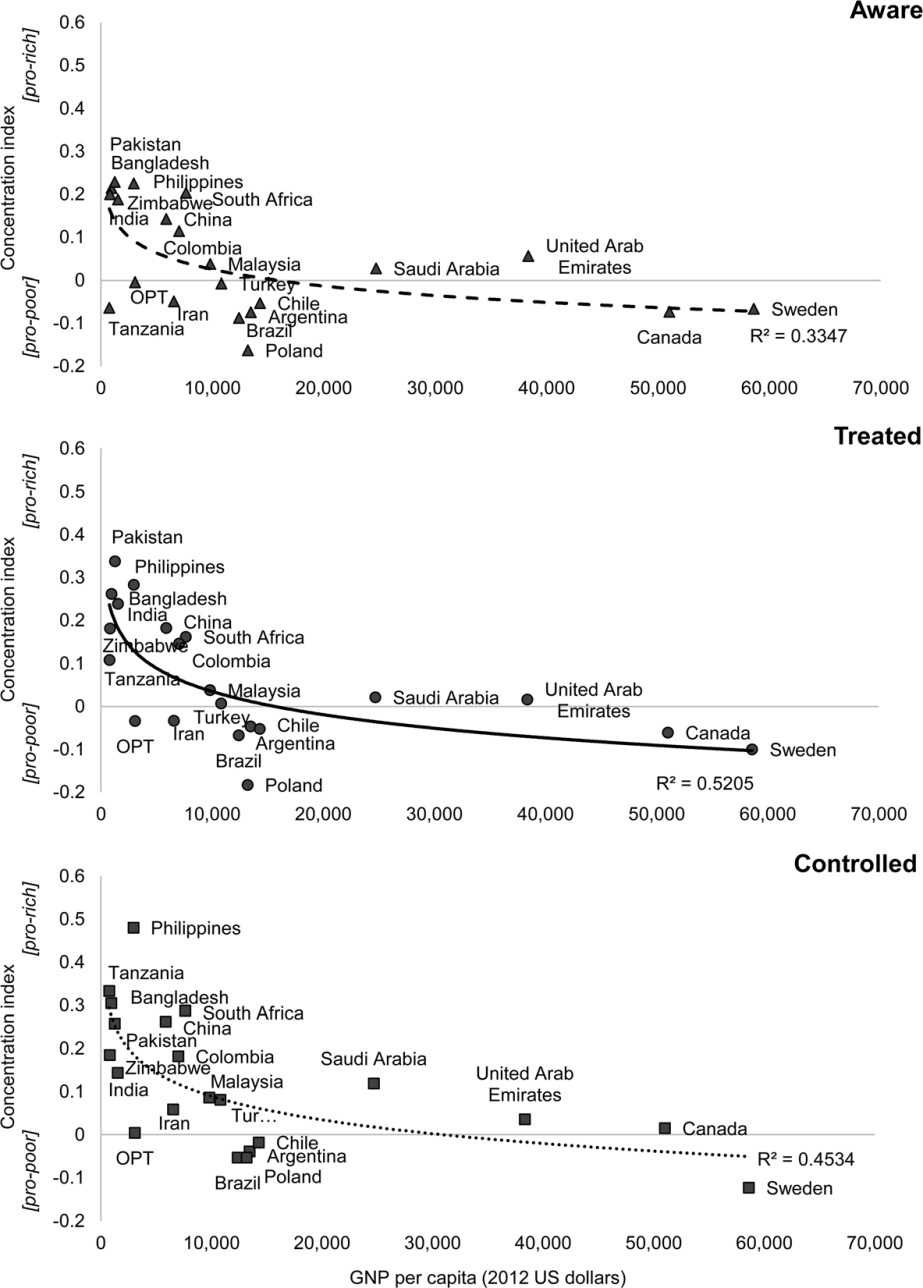
Association between concentration indices for hypertension awareness, treatment and control, and economic development measured as GNP per capita (2012 US dollars)

It might also be expected that inequalities would be greater in poor countries, which tend to have weaker health systems. Figure 3 summarizes the association between concentration indices for each hypertension management outcome and Gross National Product. For each outcome there is more variation in the levels of inequality among poorer countries, with some, such as the Philippines, having a very unequal distribution on all measures while others are much more equal. In Tanzania this is because awareness, treatment and control are poor for all groups. However, this is not the case everywhere and some, such as Iran and OPT, achieve similar outcomes for all. In rich countries the distribution is fairly similar for all groups or even pro-poor.

## Discussion

### Summary of findings

We have previously shown that there is sub-optimal control of hypertension in countries at all economic levels [3]. This paper confirms that, while there are gaps along the entire management pathway, the greatest problems in most high- and middle-income countries lie in awareness and control. Once a patient is diagnosed, they are very likely to be treated but not necessarily controlled. In contrast, in most of the low-income countries there tends to be steady attrition from awareness through treatment to control. What this study adds is that the gaps from detection through treatment to control vary among people with different levels of material resources within each country and within groups of countries at similar levels of development. This emphasizes the importance of country-specific information that can inform the national policies needed to implement universal health coverage, now a target in the 2015 Sustainable Development Goals [25].

Some countries, such as Iran or OPT, exhibit relatively equitable outcomes. Others, such as the Philippines, Colombia, India and Pakistan, do not. However, both levels and distribution of outcomes are important; Tanzania and Zimbabwe achieve very poor levels of control, but for everyone. Finally, Canada, Sweden and Poland exhibit distributions that are actually pro-poor.

### Limitations

Other international comparative studies of hypertension management outcomes exist [4, 26], but PURE is unique in its geographical reach, both in including countries at all levels of development, and in the detail collected on individuals and the communities they live in. However, it is subject to a number of limitations. In addition to the recognized biases associated with using self-reported data, PURE’s design as a cohort study necessitated inclusion of subjects in communities who could feasibly and affordably be followed up over many years. Hence, the sampling framework in each country was not designed to be nationally representative, and sample sizes were relatively modest in some countries, such as Zimbabwe. Whilst analysis of the PURE household sample has showed good concordance with the national age, sex, urban/rural, education, and mortality profiles of the study countries, suggesting that there was no systemic bias in data collection [10], caution is still warranted when interpreting and extrapolating our findings to national level.

Also, the individual element reported here was limited to subjects aged 35 to 70, as they are at highest risk of cardiovascular disease. This means that our estimates of hypertension prevalence, its detection, treatment and control will be higher than those derived from individual surveys of people at all ages. Some of the most marginalized groups, such as migrant workers, will also have been missed. As with all research on inequalities, it is important to recognize the scope for artefacts where sampling methods exclude those at the extremes of the distribution, although considerable care was taken when recruiting communities to achieve representation and avoid this problem.

The asset-based wealth index, while facilitating comparisons across such a diverse range of countries, is only one approach to measuring inequalities and is subject to certain limitations. We have also adopted a parsimonious approach when estimating concentration indices, avoiding over-adjustment for other variables. Although such variables may be important determinants of inequalities, examinations of their effects would be best studied when limited to a single country or to a few similar ones as any determinant of inequality is, to some extent, context specific, such as membership of a particular ethnic group.

Notwithstanding these limitations, the data presented here provide important new information on the scale and nature of inequalities at each stage of the hypertension management pathway, presenting the data by individual country rather than in groups within the same category of economic development. This provides a much more finely grained picture, highlighting important differences in otherwise similar countries. This is critical, given that health policy development takes place at the national level.

### Comparisons with other research

Our findings are broadly consistent with the existing, albeit somewhat fragmentary research, looking primarily at differences in prevalence of hypertension or adherence to treatment, with few international comparative studies and none, to our knowledge, that look at inequalities using comparable measures, such as the concentration index. Thus, we found 92% of Canadians aware of hypertension were being treated, compared with 82% in the Canadian Community Health Survey [27]. While the rates of awareness, treatment and control in Sweden were substantially lower than the other high-income PURE countries, they were similar to those in an earlier study that used data from the WHO MONICA (Multinational MONItoring of trends and determinants in CArdiovascular disease) Project [28]. In Iran, data from the National Surveillance of Risk Factors for NCDs also showed a similarly large attrition in the pathway of hypertensive individuals from detection through treatment to control (33% treated and 12% controlled, respectively, compared with 51 to 18% in our study) [29]. A recent paper combined data from SAGE (WHO Study on global AGEing and adult health) and COURAGE (Collaborative Research on Ageing in Europe), with data on hypertension prevalence by household wealth [5]. Among the countries in common with PURE, similar levels of hypertension awareness and control were reported: in China, 43% were aware and 8% controlled vs. 42 and 8% in our study; in India, 38% aware and 14% controlled vs. 42 and 14% in our study; and in South Africa, 38% aware and 8% controlled vs. 31 and 6% in our study.

Studies examining wealth-related inequalities are much fewer in number, however the pro-rich inequalities observed for detection and control observed in our study were consistent with previous findings in China, India and South Africa [5]; as were other study findings regarding hypertension treatment rates in Brazil [30], China [31], Iran [29] and Tanzania [32]. Finally, a recent Canadian study of adults using national data found no evidence for income-related inequality with respect to hypertension detection or control [33], while we observed a small, albeit significant, pro-poor distribution for detection but not control.

### Interpretation

The most important message from this study is that generalizations based on research in a few countries must be cautious. We describe considerable diversity in the patterns observed even in countries at similar levels of development, both at what point on the clinical pathway the problems are greatest and in how detection, treatment and control vary by household wealth. Consequently, further research within individual countries is needed to understand better how characteristics of national health systems influence treatment seeking behavior for hypertension. Studies such as this one can only be a first step in developing appropriate policies.

Nonetheless, the observed gaps and inequalities still highlight the importance of ensuring universal access to affordable, efficient and contextually appropriate methods to detect hypertension in middle age, and when detected, to provide continuous access to skilled health workers and treatment, including medications. As such, good population-level hypertension control is unlikely to be achieved unless approaches to universal health care include mechanisms to ensure access to both primary care and essential drugs for all.

As alluded to above, further research is being taken forward within PURE in the form of detailed health system analyses. Those completed so far reveal complex pictures relating to both demand for treatment, influenced by cost of drugs and by how hypertension – a chronic, asymptomatic, but hazardous condition – is conceptualized, and by supply of trained health professionals and medicines [34, 35]. Other data from the Environmental Profile of a Community’s Health study (EPOCH), nested within PURE, also provide insights. Among the upper-middle income countries, treatment rates were highest in Brazil and Poland, where all pharmacies surveyed in PURE communities had four common CVD medicines in stock and the median cost, as a percentage of ability, to pay was 3 and 1% respectively. On the other hand, the lowest treatment rates were in Malaysia and South Africa, where availability was, respectively, 37 and 33% and the cost as a percentage of ability to pay was 9 and 32% [36]. This has clear consequences for inequalities in treatment and control, particularly as effective blood pressure control typically requires use of two or more medicines [37]. Thus, wealth-related inequality in hypertension control was pro-rich in Malaysia and South Africa, and in many other lower middle- and low-income countries. In contrast, it was relatively equitable in Iran, where access to affordable medicines is good [38].

Some other observations are possible based on knowledge of the health systems involved. In general, the percentage of participants aware of hypertension and on treatment tends to be higher in countries with universal health care, but only if it renders long-term medication affordable. Universal health care should also reduce inequalities where levels of coverage are high. Brazil has implemented wide-ranging healthcare reforms, including provision of common medications for free, with one survey finding that over 90% of users of the public system, the Family Health Strategy, who were aware of hypertension were taking medicine [39]. Yet, while China has three insurance schemes that also provide some degree of coverage for most of the population, medicines are paid for out of pocket and can be expensive [40]. In contrast, though India has yet to implement universal health coverage, it has a thriving domestic generics industry with high levels of availability, but affordability is poor [36].

## Conclusion

The burden of hypertension is high in countries at all levels of development but some are able to deliver better and more equitable care. However, there is no consistent pattern. This emphasizes the need for individually tailored national solutions, an approach incorporated within the World Heart Federation hypertension roadmap [41], which sets out practical steps to improve hypertension control for all.

## Additional files

Additional file 1: Timing of data collection in each country. (PDF 14 kb)
Additional file 2: PURE study participant selection methodology. (PDF 62 kb)
Additional file 3 National representativeness of the PURE samples. (PDF 38 kb)
Additional file 4: PURE study data collection on CVD risk factors (PDF 36 kb)
Additional file 5: Additional details on the asset-based wealth index used in the PURE study. (PDF 42 kb)
Additional file 6: Hypertension prevalence, awareness, treatment and control in the PURE cohort using higher threshold definition of hypertension, by country (ordered by 2006 GDP). (PDF 103 kb)
Additional file 7: Figure S1. Patterns of adjusted hypertension prevalence with 95% confidence intervals within PURE cohorts by wealth quintile, and standardized hypertension concentration indices with 95% confidence intervals, by country (ordered by 2006 GDP). (PDF 15 kb)
Additional file 8: Figure S2. Adjusted prevalence of hypertension awareness, treatment and control with 95% confidence intervals within PURE cohorts, by wealth quintile and country (ordered by 2006 GDP). (PDF 26 kb)
Additional file 9: Figure S3. Magnitude of wealth-related inequalities in hypertension prevalence, awareness, treatment and control measured by slope index of inequality with 95% confidence interval, by country (ordered by 2006 GDP). (PDF 13 kb)
Additional file 10: Acknowledgments and funding sources. (PDF 76 kb)

## Abbreviations

CI: Confidence interval
COURAGE: Collaborative research on ageing in Europe
CVD: Cardiovascular diseases
DHS: Demographic and health surveys
EPOCH: Environmental profile of a community’s health study
GDP: Gross domestic product
GNP: Gross national product
MONICA: Monitoring of trends and determinants in cardiovascular disease project
NCD: Non-communicable disease
OPT: Occupied Palestinian territories
PURE: Prospective urban rural epidemiology study
SAGE: Study on global ageing and adult health
SD: Standard deviation
SES: Socio-economic status
UAE: United Arab Emirates
WHO STEPS: WHO stepwise approach to surveillance
WHO: World Health Organization
