# Supplementary material for: Wealth and cardiovascular health: a cross-sectional study of wealth-related inequalities in the awareness, treatment and control of hypertension in high-, middle- and low-income countries
Source: Int J Equity Health. 2016 Dec 8;15:199. doi: 10.1186/s12939-016-0478-6 (PMC5146857; doi:10.1186/s12939-016-0478-6)
Supplement: Additional file 1: — Timing of data collection in each country. (PDF 14 kb) [file 12939_2016_478_MOESM1_ESM.pdf]

Appendix S1: Timing of data collection in each country

2006 World Bank Country Income Group	Country	Data collection dates
High income	Canada	2006-2009
	Sweden	2005-2009
	United Arab Emirates	2006-2009
	Saudi Arabia	2012-2014
Upper-middle income	Argentina	2006-2009
	Brazil	2005-2009
	Chile	2006-2009
	Malaysia	2007-2009
	Poland	2007-2009
	South Africa	2005-2011
	Turkey	2008-2009
Lower-middle income	China	2005-2010
	Philippines	2013-2014
	Colombia	2005-2009
	Iran	2006-2009
	Occupied Palestinian Territory	2012-2013
Low income	Bangladesh	2008
	India	2002-2007
	Pakistan	2009-2011
	Zimbabwe	2006-2007
	Tanzania	2012-2014
