# Supplementary material for: Wealth and cardiovascular health: a cross-sectional study of wealth-related inequalities in the awareness, treatment and control of hypertension in high-, middle- and low-income countries
Source: Int J Equity Health. 2016 Dec 8;15:199. doi: 10.1186/s12939-016-0478-6 (PMC5146857; doi:10.1186/s12939-016-0478-6)
Supplement: Additional file 2: — PURE study participant selection methodology. (PDF 62 kb) [file 12939_2016_478_MOESM2_ESM.pdf]

Appendix S2: PURE study participant selection methodology (Teo et al. 2009)

Selection of Countries

The choice and number of countries selected in PURE reflects a balance between involving a large number of communities in countries at different economic levels, with substantial heterogeneity in social and economic circumstances and policies, and the feasibility of centers to successfully achieve long-term follow-up. Thus, PURE included sites in which investigators are committed to collecting good-quality data for a low-budget study over the planned 10-year follow-up period and did not aim for a strict proportionate sampling of the entire world.

Selection of Communities

Within each country, urban and rural communities were selected based on broad guidelines. A common definition for "community" that is applicable globally is difficult to establish. In PURE, a community was defined as a group of people who have common characteristics and reside in a defined geographic area. A city or large town was not usually considered to be a single community, rather communities from low-, middle-, and high-income areas were selected from sections of the city and the community area defined according to a geographical measure (e.g. a set of contiguous postal code areas or a group of streets or a village). The primary sampling unit for rural areas in many countries was the village. The reason for inclusion of both urban and rural communities is that for many countries, urban and rural environments exhibit distinct characteristics in social and physical environment, and hence, by sampling both, we ensured considerable variation in societal factors across PURE communities.

The number of communities selected in each country varied, with the aim to recruit communities with substantial heterogeneity in social and economic circumstances balanced against the capacity of local investigators to maintain follow-up. In some countries (e.g. India, China, Canada, and Colombia), communities from several states/provinces were included to capture regional diversity, in policy, socioeconomic status, culture, and physical environment. In other countries (e.g., Iran, Poland, Sweden, and Zimbabwe), fewer communities were selected.

Selections of Households and Individuals

Within each community, sampling was designed to achieve a broadly representative sample of that community of adults aged between 35 and 70 years. The choice of sampling frame within each center was based on both "representativeness" and feasibility of long-term follow-up, following broad study guidelines. Once a community was identified, where possible, common and standardized approaches were applied to the enumeration of households, identification of individuals, recruitment procedures, and data collection.

The method of approaching households differed between regions. For example, in rural areas of India and China, a community announcement was made to the village through contact of a community leader, followed by in-person door-to-door visits of all households. In contrast in Canada, initial contact was by mail followed by telephone inviting members of the households to a central clinic. Households were

eligible if at least 1 member of the household was between the ages of 35 and 70 years and the household members intended to continue living in their current home for a further 4 years.

For each approach, at least 3 attempts at contact were made. All individuals within these households between 35 and 70 years providing written informed consent were enrolled. When an eligible household or eligible individual in a household refused to participate, demographics and self-reported data about CVD risk factors, education, and history of CVD, cancers and deaths in the households within the two previous years were recorded.

To ensure standardization and high data quality, we used a comprehensive operations manual, training workshops, DVDs, regular communication with study personnel and standardized report forms. We entered all data in a customized database programmed with range and consistency checks that was transmitted electronically to the Population Health Research Institute in Hamilton (Ontario, Canada) where further quality checks were implemented.

Guidelines for Selection of Countries, Communities, Households, and Individuals recruited to PURE

Countries

1. High-income countries, middle-income countries, and low-income countries, with the bulk of the recruitment from low- and middle-income regions.
2. Committed local investigators with experience in recruiting for population studies.

Communities

1. Select both urban and rural communities. Use the national definition of the country to determine urban and rural communities.
2. Select rural communities that are isolated (distance of >50 km or lack easy access to commuter transportation) from urban centers. However, consider ability to process bloods samples, e.g., villages in rural developing countries should be within 45-min drive of an appropriate facility.
3. Define community to a geographical area, e.g., using postal codes, catchment area of health service/clinics, census tracts, areas bordered by specific streets or natural borders such as a river bank.
4. Consider feasibility for long-term follow-up, e.g., for urban communities, choose sites that have a stable population such as residential colonies related to specific work sites in developing countries. In rural areas, choose villages that have a stable population. Villages at greater distance from urban centers are less susceptible to large migration to urban centers.
5. Enlist a community organization to facilitate contact with the community, e.g., in urban areas, large employers (government and private), insurance companies, clubs, religious organizations, clinic or hospital service regions. In rural areas, local authorities such as priests or community elders, hospital or clinic, village leader, or local politician.

Individuals

1. Broadly representative sampling of adults 35 to 70 years within each community unit.
2. Consider feasibility for long-term follow-up when formulating community sampling framework, e.g., small percentage random samples of large communities may be more difficult to follow-up because they are dispersed by distance. In rural areas of developing countries that are not connected by telephone, it may be better to sample entire community (i.e., door-to-door systematic sampling).
3. The method of approach of households/individuals may differ between sites. In MIC and HIC, mail, followed up by phone contact may be the practical first means of contact. In LIC, direct household contact through household visits may be the most appropriate means of first contact.
4. Once recruited, all individuals are invited to a study clinic to complete standardized questionnaires and have a standardized set of measurements (note that in Bangladesh, questionnaires and measurements were completed during household visits).
