# Supplementary material for: Wealth and cardiovascular health: a cross-sectional study of wealth-related inequalities in the awareness, treatment and control of hypertension in high-, middle- and low-income countries
Source: Int J Equity Health. 2016 Dec 8;15:199. doi: 10.1186/s12939-016-0478-6 (PMC5146857; doi:10.1186/s12939-016-0478-6)
Supplement: Additional file 3 — National representativeness of the PURE samples. (PDF 38 kb) [file 12939_2016_478_MOESM3_ESM.pdf]

Appendix S3: National representativeness of the PURE samples (Corsi et al. 2013)

The PURE household population compared to national statistics had more women (sex ratio 95.1 men per 100 women vs 100.3) and was older (33.1 years vs 27.3), although age had a positive linear relationship between the two data sources (Pearson's r = 0.92). PURE was 59.3% urban compared to an average of 63.1% in participating countries. The distribution of education was less than 7% different for each category, although PURE households typically had higher levels of education. For example, 37.8% of PURE household members had completed secondary education compared to 31.3% in the national data. Age-adjusted annual mortality rates showed positive correlation for men (r = 0.91) and women (r = 0.92) but were lower in PURE compared to national statistics (7.9 per 1000 vs 8.7 for men; 6.7 vs 8.1 for women).
