# Supplementary material for: Wealth and cardiovascular health: a cross-sectional study of wealth-related inequalities in the awareness, treatment and control of hypertension in high-, middle- and low-income countries
Source: Int J Equity Health. 2016 Dec 8;15:199. doi: 10.1186/s12939-016-0478-6 (PMC5146857; doi:10.1186/s12939-016-0478-6)
Supplement: Additional file 4: — PURE study data collection on CVD risk factors (PDF 36 kb) [file 12939_2016_478_MOESM4_ESM.pdf]

Appendix S4: PURE study data collection on CVD risk factors (Yusuf et al. 2004)

Following the procedures described in the INTERHEART study, standardized questionnaires were used to collect participant information about demographic factors, socioeconomic status (education, household assets), lifestyle (smoking, leisure time, physical activity, alcohol consumption and dietary patterns), personal and family history of cardiovascular disease, and risk factors (hypertension, diabetes mellitus). Height, weight, waist and hip circumferences, blood pressure and heart rate were determined by a standardized protocol. Waist and hip circumferences were measured with a non-stretchable standard tape measure: waist measurements were obtained over the unclothed abdomen at the narrowest point between the costal margin and iliac crest, and hip circumferences over light clothing at the level of the widest diameter around the buttocks.
