# Supplementary material for: Wealth and cardiovascular health: a cross-sectional study of wealth-related inequalities in the awareness, treatment and control of hypertension in high-, middle- and low-income countries
Source: Int J Equity Health. 2016 Dec 8;15:199. doi: 10.1186/s12939-016-0478-6 (PMC5146857; doi:10.1186/s12939-016-0478-6)

Appendix S5: Additional details on the asset-based wealth index used in the PURE study

O'Donnell and Wagstaff note that concentration index is sensitive to the choice of wealth variable and this choice may have consequences when ranking countries by levels of inequality.(O'Donnell and Wagstaff 2008) Thus we do not rank countries. Our choice of wealth variable is limited by what data could feasibly be collected in the selected study countries. Given that most are low- and middle-income countries, it is recognized that an asset-based wealth index may be the best approach. The key point is that the wealth variable must be the same across all countries to conduct cross-country comparisons.(Vyas and Kumaranayake 2006)

With Principle Components Analysis (PCA), a number of issues could arise, including 'clumping' if components do not sufficiently differentiate across households.(Vyas and Kumaranayake 2006)

Another limitation is the lack of any other micro data collected in all countries that could be used as an alternative measure of household wealth to use and check if our findings are robust. This is a limitation of the study. However, PCA methods have been extensively used and validated, but may be less 'good' in HICs. The distribution of the asset-based wealth index variable by country, using a box and whisker plot, is shown below.

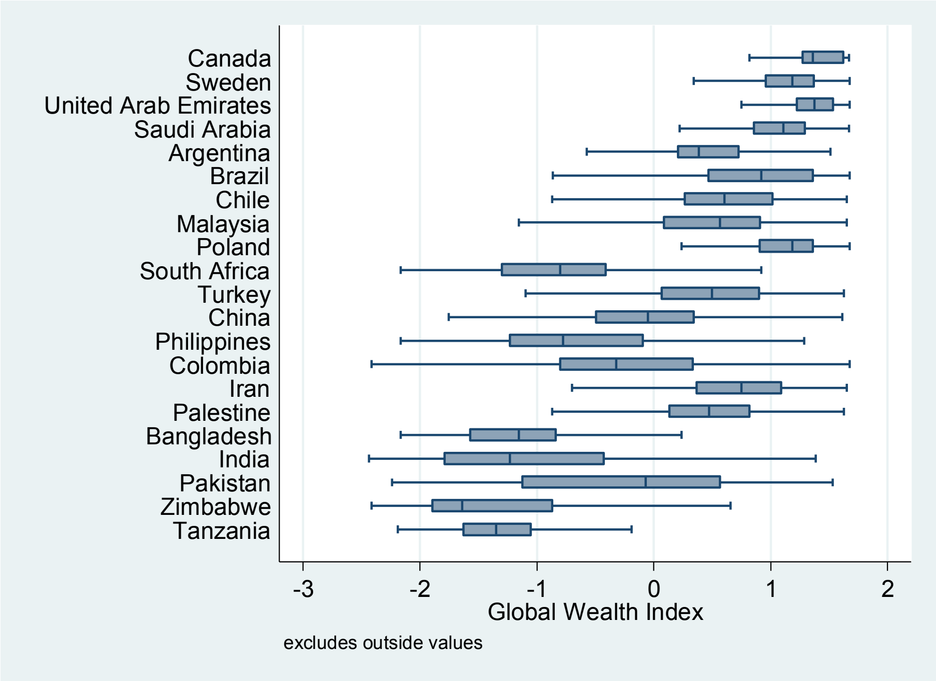

Supplement: Additional file 5: — Additional details on the asset-based wealth index used in the PURE study. (PDF 42 kb) [file 12939_2016_478_MOESM5_ESM.pdf]
