# Supplementary material for: Wealth and cardiovascular health: a cross-sectional study of wealth-related inequalities in the awareness, treatment and control of hypertension in high-, middle- and low-income countries
Source: Int J Equity Health. 2016 Dec 8;15:199. doi: 10.1186/s12939-016-0478-6 (PMC5146857; doi:10.1186/s12939-016-0478-6)
Supplement: Additional file 6: — Hypertension prevalence, awareness, treatment and control in the PURE cohort using higher threshold definition of hypertension, by country (ordered by 2006 GDP). (PDF 103 kb) [file 12939_2016_478_MOESM6_ESM.pdf]

Appendix S6: Hypertension prevalence, awareness, treatment and control in the PURE cohort using higher threshold definition of hypertension, by country (ordered by 2006 GDP)

	Participants				Hypertensive participants								
Country	Number	No. with Hypertension (%) [95%CI]			No. Aware (%) [95%CI]			No. Treated (%) [95%CI]			No. Controlled (%) [95%CI]		
Canada	10412	2395	(23.0)	[21.4-24.7]	2036	(85.0)	[83.5-86.4]	2014	(84.1)	[82.5-85.6]	1691	(70.6)	[68.5-72.6]
Sweden	4150	1042	(25.1)	[23.4-26.9]	649	(62.3)	[56.7-67.6]	584	(56.0)	[49.4-62.4]	368	(35.3)	[29.1-42.0]
UAE	918	308	(33.6)	[30.6-36.7]	242	(78.6)	[73.6-82.8]	236	(76.6)	[71.5-81.0]	158	(51.3)	[45.7-56.9]
Saudi Arabia	1729	346	(20.0)	[17.9-22.4]	312	(90.2)	[85.9-93.2]	306	(88.4)	[84.0-91.8]	265	(76.6)	[70.1-82.1]
Argentina	7497	2455	(32.7)	[31.3-34.2]	1972	(80.3)	[77.8-82.6]	1881	(76.6)	[74.0-79.1]	1258	(51.2)	[47.9-54.6]
Brazil	5581	2128	(38.1)	[35.7-40.6]	1844	(86.7)	[85.3-87.9]	1805	(84.8)	[83.4-86.2]	1254	(58.9)	[56.9-61.0]
Chile	3270	1031	(31.5)	[28.4-34.8]	825	(80.0)	[74.3-84.7]	781	(75.8)	[68.7-81.6]	597	(57.9)	[48.5-66.7]
Malaysia	11825	3313	(28.0)	[26.0-30.1]	2404	(72.6)	[68.9-76.0]	2209	(66.7)	[62.4-70.7]	1449	(43.7)	[39.6-48.0]
Poland	2029	923	(45.5)	[38.1-53.1]	708	(76.7)	[57.4-88.9]	686	(74.3)	[58.0-85.9]	405	(43.9)	[33.1-55.3]
South Africa	3252	1127	(34.7)	[24.6-46.3]	537	(47.6)	[34.5-61.2]	551	(48.9)	[40.2-57.6]	250	(22.2)	[17.0-28.3]
Turkey	4060	1030	(25.4)	[23.3-27.6]	844	(81.9)	[78.3-85.1]	789	(76.6)	[72.7-80.1]	575	(55.8)	[50.1-61.4]
China	46751	10910	(23.3)	[21.6-25.2]	7330	(67.2)	[61.8-72.1]	6466	(59.3)	[53.1-65.1]	3752	(34.4)	[28.3-41.0]
Philippines	1671	594	(35.5)	[24.5-48.3]	435	(73.2)	[45.1-90.1]	389	(65.5)	[33.9-87.5]	234	(39.4)	[17.9-65.9]
Colombia	7506	1763	(23.5)	[21.9-25.1]	1365	(77.4)	[74.6-80.0]	1288	(73.1)	[70.0-75.9]	870	(49.3)	[45.5-53.2]
Iran	6013	928	(15.4)	[13.4-17.7]	786	(84.7)	[78.5-89.4]	758	(81.7)	[75.8-86.4]	552	(59.5)	[49.8-68.4]
OPT	1563	387	(24.8)	[22.2-27.5]	328	(84.8)	[78.7-89.3]	330	(85.3)	[79.6-89.6]	232	(59.9)	[51.4-67.9]
Bangladesh	2747	598	(21.8)	[19.4-24.3]	215	(36.0)	[29.5-43.0]	167	(27.9)	[22.6-33.9]	82	(13.7)	[10.1-18.4]
India	27458	4533	(16.5)	[14.9-18.3]	3101	(68.4)	[63.6-72.9]	2784	(61.4)	[56.5-66.1]	2045	(45.1)	[40.2-50.2]
Pakistan	1294	244	(18.9)	[13.6-25.6]	178	(73.0)	[62.4-81.4]	157	(64.3)	[50.9-75.8]	118	(48.4)	[41.9-54.9]
Zimbabwe	822	292	(35.5)	[20.3-54.4]	187	(64.0)	[41.4-81.8]	118	(40.4)	[27.3-55.1]	58	(19.9)	[12.8-29.5]
Tanzania	1071	182	(17.0)	[12.5-22.7]	28	(15.4)	[10.1-22.7]	10	(5.5)	[3.5-8.6]	3	(1.6)	[0.6-4.8]
Total	151619	36529	(24.1)		26326	(72.1)		24309	(66.5)		16216	(44.4)	

OPT: Occupied Palestinian Territory; UAE: United Arab Emirates; GDP: gross domestic product; CI: confidence interval. Note: *Hypertension higher threshold definition: systolic blood pressure ≥160 mm Hg or diastolic blood pressure ≥100 mm Hg or who report currently taking medication.
