# Supplementary material for: Wealth and cardiovascular health: a cross-sectional study of wealth-related inequalities in the awareness, treatment and control of hypertension in high-, middle- and low-income countries
Source: Int J Equity Health. 2016 Dec 8;15:199. doi: 10.1186/s12939-016-0478-6 (PMC5146857; doi:10.1186/s12939-016-0478-6)

Appendix S7: Patterns of adjusted hypertension prevalence with 95% confidence intervals within PURE cohorts by wealth quintile, and standardised hypertension concentration indices with 95% confidence intervals, by country (ordered by 2006 GDP)

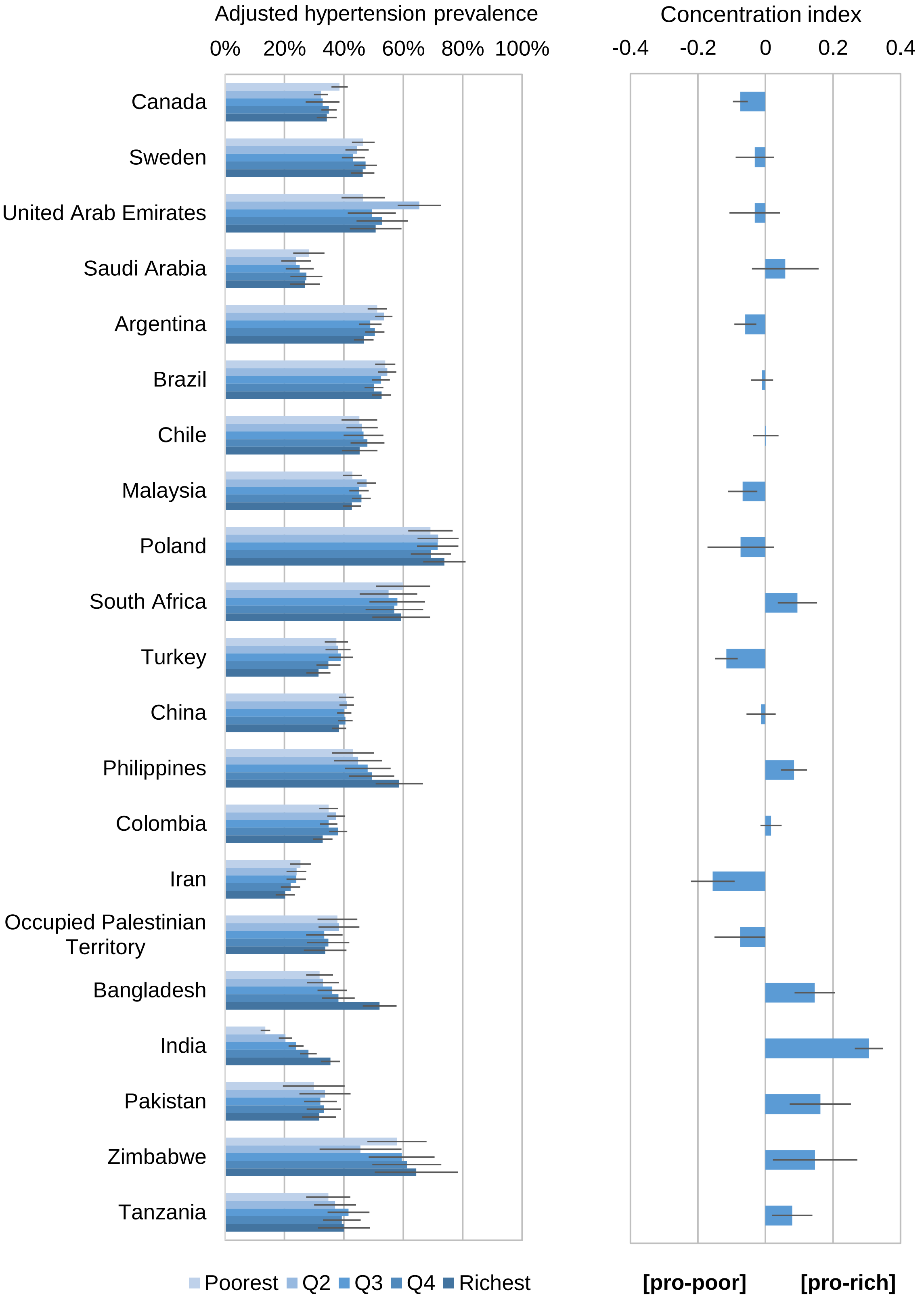

Supplement: Additional file 7: Figure S1. — Patterns of adjusted hypertension prevalence with 95% confidence intervals within PURE cohorts by wealth quintile, and standardized hypertension concentration indices with 95% confidence intervals, by country (ordered by 2006 GDP). (PDF 15 kb) [file 12939_2016_478_MOESM7_ESM.pdf]
