# Supplementary material for: Wealth and cardiovascular health: a cross-sectional study of wealth-related inequalities in the awareness, treatment and control of hypertension in high-, middle- and low-income countries
Source: Int J Equity Health. 2016 Dec 8;15:199. doi: 10.1186/s12939-016-0478-6 (PMC5146857; doi:10.1186/s12939-016-0478-6)

Appendix S8: Adjusted prevalence of hypertension awareness, treatment and control with 95% confidence intervals within PURE cohorts, by wealth quintile and country (ordered by 2006 GDP)

(Note: *Adjusted estimates for hypertension control by wealth quintile in Tanzania could not be obtained due to the low number of positive outcomes.)

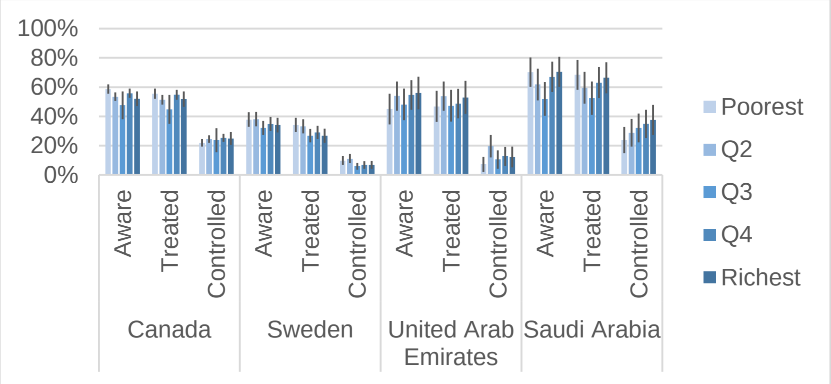

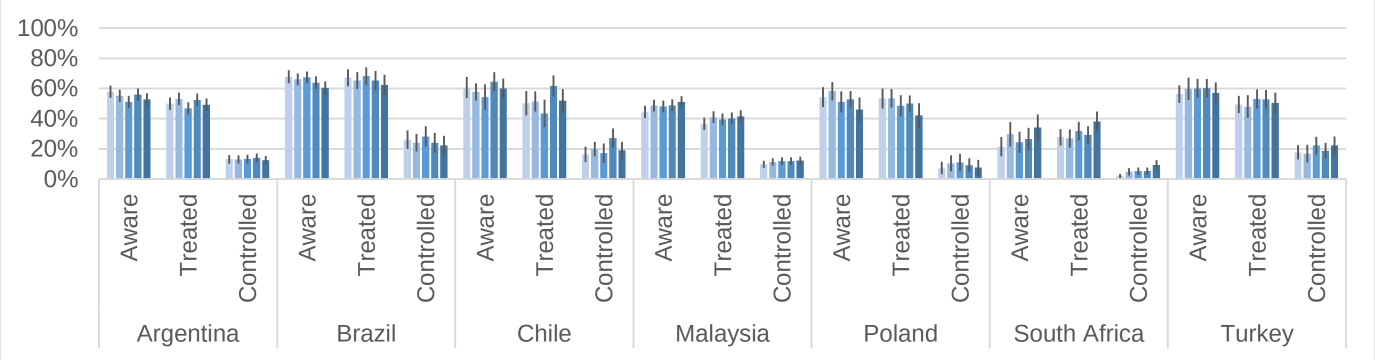

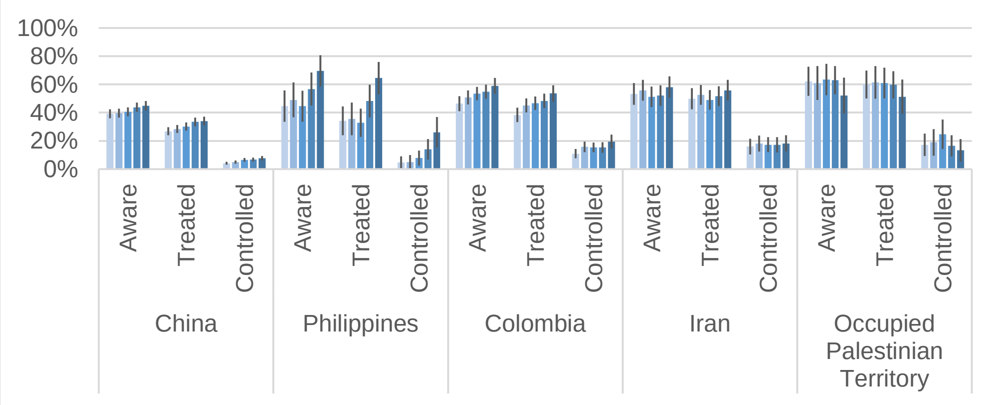

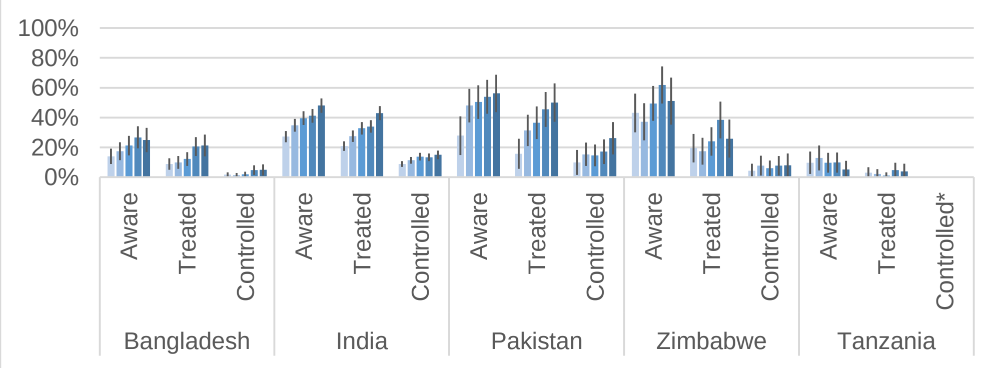

Supplement: Additional file 8: Figure S2. — Adjusted prevalence of hypertension awareness, treatment and control with 95% confidence intervals within PURE cohorts, by wealth quintile and country (ordered by 2006 GDP). (PDF 26 kb) [file 12939_2016_478_MOESM8_ESM.pdf]
