# Supplementary material for: Wealth and cardiovascular health: a cross-sectional study of wealth-related inequalities in the awareness, treatment and control of hypertension in high-, middle- and low-income countries
Source: Int J Equity Health. 2016 Dec 8;15:199. doi: 10.1186/s12939-016-0478-6 (PMC5146857; doi:10.1186/s12939-016-0478-6)
Supplement: Additional file 10: — Acknowledgments and funding sources. (PDF 76 kb) [file 12939_2016_478_MOESM10_ESM.pdf]

Appendix S10: Acknowledgments and funding sources

PURE Project Office Staff, National Coordinators, Investigators and Key Staff

Project office (Population Health Research Institute, Hamilton Health Sciences and McMaster University, Hamilton, Canada): S Yusuf* (Principal Investigator).

S Rangarajan (Project Manager); K K Teo, C K Chow, M O'Donnell, A Mente, D Leong, A Smyth, P Joseph, S Islam (Statistician), M Zhang (Statistician), W Hu (Statistician), C Ramasundarahettige (Statistician), G Wong (Statistician), L Dayal, A Casanova, M Dehghan (Nutritionist), G Lewis, J DeJesus, P Mackie, SL Chin, D Hari, L Farago, I Kay, D Agapay, R Solano, S Ramacham, N Kandy, J Rimac, S Trottier, W ElSheikh, M Mustaha, T Tongana, N Aoucheva, J Swallow, E Ramezani, A Aliberti, J Lindeman

Core Laboratories: M McQueen, K Hall, J Keys (Hamilton), X Wang (Beijing, China), J Keneth, A Devanath (Bangalore, India).

ARGENTINA: R Diaz*; A Orlandini, B Linetsky, S Toscanelli, G Casaccia, JM Maini Cuneo; **BANGLADESH:** O Rahman*, R Yusuf, AK Azad, KA Rabbani, HM Cherry, A Mannan, I Hassan, AT Talukdar, RB Tooheen, MU Khan, M Sintaha, T Choudhury, R Haque, S Parvin; **BRAZIL:** A Avezum*, GB Oliveira, CS Marcilio, AC Mattos; **CANADA:** K Teo*, S Yusuf*, J Dejesus, D Agapay, T Tongana, R Solano, I Kay, S Trottier, J Rimac, W Elsheikh, L Heldman, E Ramezani, G Dagenais, P Poirier, G Turbide, D Auger, A LeBlanc De Bluts, MC Proulx, M Cayer, N Bonneville, S Lear, D Gasevic, E Corber, V de Jong, I Vukmirovich, A Wielgosz, G Fodor, A Pipe, A Shane; **CHILE:** F Lanas*, P Seron, S Martinez, A Valdebenito, M Oliveros; **CHINA:** Li Wei*, Liu Lisheng*, Chen Chunming, Wang Xingyu, Zhao Wenhua, Zhang Hongye, JiaXuan, Hu Bo, Sun Yi, Bo Jian, Zhao Xiuwen, Chang Xiaohong, Chen Tao, Chen Hui, Chang Xiaohong, Deng Qing, Cheng Xiaoru, Deng Qing, He Xinye, Hu Bo, JiaXuan, Li Jian, Li Juan,Liu Xu, Ren Bing, Sun Yi, Wang Wei, Wang Yang, Yang Jun, Zhai Yi, Zhang Hongye, Zhao Xiuwen,Zhu Manlu, Lu Fanghong, Wu Jianfang, Li Yindong, Hou Yan, Zhang Liangqing, Guo Baoxia, Liao Xiaoyang, Zhang Shiying, BianRongwen, TianXiuzhen, Li Dong, Chen Di, Wu Jianguo, Xiao Yize, Liu Tianlu, Zhang Peng, Dong Changlin, Li Ning, Ma Xiaolan, Yang Yuqing, Lei Rensheng, Fu Minfan, He Jing, Liu Yu, Xing Xiaojie, Zhou Qiang, ; **COLOMBIA:** P Lopez-Jaramillo*, PA Camacho Lopez, R Garcia, LJA Jurado, D Gómez-Arbeláez, JF Arguello, R Dueñas, S Silva, LP Pradilla, F Ramirez, DI Molina, C Cure-Cure, M Perez, E Hernandez, E Arcos, S Fernandez, C Narvaez, A Sotomayor, H Garcia, G Sanchez, T David, A Rico; **INDIA:** P Mony *, M Vaz*, A V Bharathi, S Swaminathan, K Shankar AV Kurpad, KG Jayachitra, N Kumar, HAL Hospital, V Mohan, M Deepa, K Parthiban, M Anitha, S Hemavathy, T Rahulashankiruthiyayan, D Anitha, K Sridevi, R Gupta, RB Panwar, I Mohan, P Rastogi, S Rastogi, R Bhargava, R Kumar, J S Thakur, B Patro, PVM Lakshmi, R Mahajan, P Chaudary, V Raman Kutty, K Vijayakumar, K Ajayan, G Rajasree, AR Renjini, A Deepu, B Sandhya, S Asha, HS Soumya;**IRAN:** R Kelishadi*, A Bahonar, N Mohammadifard, H Heidari; **MALAYSIA:** K Yusoff*, TST Ismail, KK Ng, A Devi, NM Nasir, MM Yasin, M Miskan, EA Rahman, MKM Arsad, F Ariffin, SA Razak, FA Majid, NA Bakar, MY Yacob, N Zainon, R Salleh, MKA Ramli, NA Halim, SR Norlizan, NM Ghazali, MN Arshad, R Razali, S Ali, HR Othman, CWJCW Hafar, A Pit, N Danuri, F Basir, SNA Zahari, H Abdullah, MA Arippin, NA Zakaria, I Noorhassim, MJ Hasni, MT Azmi, MI Zaleha, KY Hazdi, AR Rizam, W Sazman, A Azman; **OCCUPIED PALESTINIAN TERRITORY:** R Khatib*, U Khammash, A Khatib, R Giacaman; **PAKISTAN:** R Iqbal*, A Afridi, R Khawaja, A Raza, K Kazmi; **PHILIPPINES:** A Dans*, HU Co, JT Sanchez, L Pudol, C Zamora-Pudol, LM Palileo-Villanueva, MR Aquino, C Abaquin, SL Pudol, ML Cabral; **POLAND:** W Zatonski*, A Szuba, K Zatonska, R Ilow[#], M Ferus, B Regulska-Ilow, D Różańska, M

Wolyniec; **SAUDI ARABIA:** KF AlHabib*, A Hersi, T Kashour, H Alfaleh, M Alshamiri, HB Altaradi, O Alnobani, A Bafart, N Alkamel, M Ali, M Abdulrahman, R Nouri; **SOUTH AFRICA:** A Kruger*, L Kruger*, H H Voster, A E Schutte, E Wentzel-Viljoen, FC Eloff, H de Ridder, H Moss, J Potgieter, AA Roux, M Watson, G de Wet, A Olckers, JC Jerling, M Pieters, T Hoekstra, T Puoane, E Igumbor, L Tsolekile, D Sanders, P Naidoo, N Steyn, N Peer, B Mayosi, B Rayner, V Lambert, N Levitt, T Kolbe-Alexander, L Ntyintyane, G Hughes, R Swart, J Fourie, M Muzigaba, S Xapa, N Gobile , K Ndayi, B Jwili, K Ndibaza, B Egbujie; **SWEDEN:** *, K Bengtsson Boström, U Lindblad, P Langkilde, A Gustavsson, M Andreasson, M Snällman, L Wirdemann, K Pettersson, E Moberg ; **TANZANIA:** K Yeates*, J Sleeth, K Kilonzo**;** **TURKEY:** A Oguz*, AAK Akalin, KBT Calik, N Imeryuz, A Temizhan, E Alphan, E Gunes, H Sur, K Karsidag, S Gulec, Y Altuntas; **UNITED ARAB EMIRATES:** AM Yusufali*, W Almahmeed, H Swidan, EA Darwish, ARA Hashemi, N Al-Khaja, JM Muscat-Baron, SH Ahmed, TM Mamdouh, WM Darwish, MHS Abdelmotagali, SA Omer Awed, GA Movahedi, F Hussain, H Al Shaibani, RIM Gharabou, DF Youssef, AZS Nawati, ZAR Abu Salah, RFE Abdalla, SM Al Shuwaihi, MA Al Omairi, OD Cadigal; R.S. Alejandrino; **ZIMBABWE:** J Chifamba*, L Gwaunza, G Terera, C Mahachi, P Murambiwa, T Machiweni, R Mapanga.

*National Coordinator

[#] Deceased

Funding sources

The UK Economic and Social Research Council funded MM, DB and BP for these analyses through a grant (number ES/L014696/1) under its Secondary Data Analysis Initiative scheme. SY is funded by the Marion Burke Chair of the Heart and Stroke Foundation of Canada.

The main PURE study and its components are funded by the Population Health Research Institute, the Canadian Institutes of Health Research, Heart and Stroke Foundation of Ontario and through unrestricted grants from several pharmaceutical companies [with major contributions from Astra Zeneca (Canada), Sanofi-Aventis (France and Canada), Boehringer Ingelheim (Germany & Canada), Servier, and GSK], and additional contributions from Novartis and King Pharma and from various national or local organizations in participating countries.

These include: Argentina: Fundacion ECLA; Bangladesh: Independent University, Bangladesh and Mitra and Associates; Brazil: Unilever Health Institute, Brazil; Canada: Public Health Agency of Canada and Champlain Cardiovascular Disease Prevention Network; Chile: Universidad de la Frontera; China: National Center for Cardiovascular Diseases; Colombia: Colciencias, Grant number:6566-04-18062 and Fundacion Oftalmologica de Santander ; India: Indian Council of Medical Research; Malaysia: Ministry of Science, Technology and Innovation of Malaysia Grant Nbr 100 - IRDC / BIOTEK 16/6/21 (13/2007), Grant Number 07-05-IFN-BPH 010, Ministry of Higher Education of Malaysia Grant Nbr 600 - RMI/LRGS/5/3 (2/2011), Universiti Teknologi MARA, Universiti Kebangsaan Malaysia (UKM-Hejim-Komuniti-15-2010); occupied Palestinian territory: the United Nations Relief and Works Agency for Palestine Refugees in the Near East (UNRWA), occupied Palestinian territory; International Development Research Centre (IDRC), Canada; Philippines: Philippine Council for Health Research & Development (PCHRD); Poland: Polish Ministry of Science and Higher Education grant Nr 290/W-PURE/2008/0, Wroclaw Medical University; Saudi Arabia: Saudi Heart

Association. The Deanship of Scientific Research at King Saud University, Riyadh, Saudi Arabia (Research group number: RG -1436-013); South Africa: The North-West University, SANPAD (SA and Netherlands Programme for Alternative Development), National Research Foundation, Medical Research Council of SA, The SA Sugar Association (SASA), Faculty of Community and Health Sciences (UWC); Sweden: AFA Insurance, Swedish Council for Working Life and Social Research, Swedish Research Council for Environment, Agricultural Sciences and Spatial Planning, Swedish Heart and Lung Foundation, Swedish Research Council, Grant from the Swedish State under (LäkarUtbildningsAvtalet), Agreement, Grant from the Västra Götaland Region (FOUU); TURKEY: Metabolic Syndrome Society, Astra Zeneca, Turkey, Sanofi Aventis, Turkey; UAE: Sheikh Hamdan Bin Rashid Al Maktoum Award For Medical Sciences and Dubai Health Authority, Dubai UAE.

The funders of the study had no role in its design, data collection, data analysis, data interpretation, or writing of the report. The corresponding and lead (MM and BP) authors had full access to all the data in the study and all authors had final responsibility for the decision to submit for publication.
